# Ecological and evolutionary implications of a mobile genetic element-rich haloarchaeon with unique osmotic resilience

**DOI:** 10.1128/spectrum.03403-25

**Published:** 2026-06-30

**Authors:** Yimin Ni, Wanling Bi, Hao Yu, Lanming Chen, Yongxin Yu, Jigang Han, Yongjie Wang

**Affiliations:** 1 College of Food Science and Technology, Shanghai Ocean University, Shanghai, China; 2 Laboratory for Marine Biology and Biotechnology, Qingdao Marine Science and Technology Center, Qingdao, China; 3 Shanghai Wildlife and Protected Natural Areas Research Center, Shanghai, China; North Carolina State University, Raleigh, North Carolina, USA

**Keywords:** horizontal gene transfer, megaplasmid, low salinity, halophilic archaeon

## Abstract

We isolated a novel halophilic archaeon, strain DSL9, representing the proposed new species *Haloliberatus hailidukes* gen. nov., sp. nov., from Dishui Lake, China. Unlike most obligate halophiles, DSL9 survives in low salinity, even distilled water, without lysis. Genomic analysis revealed dual salinity adaptation strategies: salt-in and compatible solutes, including a complete trehalose biosynthesis pathway. The strain harbors multiple plasmids, notably a 111,311 bp large plasmid (pHdsl9-3) encoding replication (Orc1/Cdc6, SSB), transcription (TFIIB), transmission (T4SS cluster, ArdC-like protein), and recombination (XerA) modules. pHdsl9-3 provides auxiliary functions such as defense, genome diversification, ion detoxification, and suggests active horizontal gene transfer. Similar elements are widespread in *Halobacteriales*, highlighting their role in haloarchaeal genetic diversity and plasticity. The encoded XerA hinted at a function beyond DNA dimer resolution, suggesting it may have been adapted by other archaeal mobile genetic elements. These findings underscore the need to investigate plasmid-driven evolution and environmental adaptation mechanisms in haloarchaea.

**IMPORTANCE:**

This study reports the isolation and characterization of DSL9, a novel halophilic archaeon from a freshwater lake. Remarkably, DSL9 defies the typical obligate halophilic lifestyle by surviving in low-salinity environments, including distilled water, without cell lysis. A key discovery is the identification of a 111,311 bp large plasmid harboring essential modules for replication, transcription, transmission, and integration. Widespread distribution of similar elements across *Halobacteriales* suggests their crucial role in haloarchaeal genetic diversity and plasticity, warranting further study of plasmid-mediated evolution and adaptation strategies.

## INTRODUCTION

Haloarchaea, a term designating members of the phylum *Halobacteriota*, primarily inhabit hypersaline environments, including salt lakes, solar salterns, and saline soils (1–3). Their ease of isolation and cultivation has established them as key model organisms in archaeal research (4). Recent metagenomic advances have expanded the taxonomic framework, with the class *Halobacteria* now encompassing 910 genomes across 19 families in the GTDB classification system (5). These extremophiles thrive in environments characterized by extreme osmotic pressure, with most exhibiting obligate halophily (3, 6). Their strict dependence on high salinity stems from fundamental physiological constraints: exposure to hypotonic conditions triggers cellular lysis and impairs protein function through charge distribution alterations (7).

To maintain cellular integrity in hypersaline habitats, haloarchaea employ two primary osmoregulatory strategies: the “salt-in” approach involving potassium accumulation and sodium exclusion, and the “compatible solute” mechanism based on organic osmolyte synthesis (7). While salinity fluctuations prove catastrophic for most obligate halophiles, causing osmotic imbalance, structural collapse, and protein denaturation (8, 9), emerging research reveals adaptive responses in some species. Recent investigations have documented survival mechanisms during hyposaline stress, including discernible physiological and morphological adaptations (10, 11), challenging previous assumptions about their environmental plasticity.

Haloarchaea exhibit another remarkable feature: their frequent encompassing of multiple plasmids, ranging from several kilobase pairs to hundreds of kilobase pairs in size (12–14). This extensive plasmid diversity makes them particularly valuable for bioengineering applications. In prokaryotes, plasmids serve as evolutionary accelerators by enabling horizontal gene transfer, enhancing genetic diversity, and facilitating rapid host adaptation to environmental changes (15). Some haloarchaeal plasmids even challenge conventional biological categorizations. For instance, the virus-like plasmid pR1SE employs unique transmission strategies that blur the distinction between plasmids and viruses (16, 17).

Further highlighting their genomic plasticity, studies have documented substantial genomic content loss in haloarchaea during laboratory cultivation, often due to the activity of insertion sequences (18–21). The integration of mobile genetic elements such as plasmids and viruses in archaea is predominantly facilitated by site-specific recombinases, with tyrosine recombinase representing one of the most extensively studied families. These enzymes play multifaceted roles in genome maintenance and plasticity, including (i) resolving dimerized DNA through precise recombination (22, 23), (ii) mediating genomic inversions that can regulate gene expression (24), and (iii) catalyzing the integration of foreign genetic material from both viral and plasmid sources (25, 26). Recent studies have further expanded our understanding of their diverse functions, revealing additional roles in genome rearrangement and stability maintenance (27).

This study reports the isolation of a remarkable halophilic archaeal strain capable of surviving extreme hyposaline stress, including exposure to distilled water. Our findings provide new perspectives to haloarchaeal adaptation through the discovery of previously uncharacterized mobile haloarchaeal large plasmids. The identification of this element suggests the existence of additional large-scale mobile genetic elements in haloarchaea that utilize XerA recombinase-mediated mechanisms. These results significantly expand our understanding of haloarchaeal genome plasticity (13, 28), revealing potential new pathways for extreme environmental adaptation through large DNA element mobilization and recombination.

## MATERIALS AND METHODS

### Isolation and cultivation of strain DSL9

The isolation site characteristics for strain DSL9 are detailed in Table 1. Water samples were processed by filtration through 0.45 μm membranes to concentrate microorganisms, followed by resuspension in distilled water. Samples were plated on solid modified growth medium (a total salinity of 18% [wt/vol] MGM) containing (per liter) 600 mL 30% synthetic seawater (30% SW), 5 g peptone, 1 g yeast extract, 15 g agar, and 5 mL 1 M Tris-HCl (pH 7.5). The 30% SW component consisted of (per liter) 240 g NaCl, 30 g MgCl₂·6H₂O, 35 g MgSO₄·7H₂O, 7 g KCl, and 5 mL 1 M Tris-HCl (pH 7.5) (29).

**TABLE 1:** Characteristics of the collected water samples

Source	Sampling depth (m)	Longitude and latitude of sampling site	pH	Salinity (%)	Total dissolved solids (ppm)
Dishui Lake, Shanghai	−1	30°53′55″ N, 121°55′25″ E	8.72 ± 0.02	0.04 ± 0.00	482 ± 2

Plates were incubated aerobically at 37°C for one month. Distinct colonies were subsequently isolated through repeated streaking on fresh MGM agar plates to ensure culture purity. For long-term preservation, isolates were stored in liquid MGM supplemented with 25% (wt/vol) glycerol at −80°C.

### Cellular characterization and phenotypic analysis

Cell morphology was examined using transmission electron microscopy (HT-7800, Hitachi, Japan). For negative staining, cells were treated with 2% phosphotungstic acid for 60 s and imaged at 80–120 kV. For ultrastructural analysis, cells were processed through (i) primary fixation with 1% osmium tetroxide (1–2 h) after PBS washes; (ii) ethanol dehydration series (30–100%) followed by acetone rinses; (iii) embedding in resin with graded infiltration (50–100%) and polymerization (70°C, 12–48 h); and (iv) ultrathin sectioning (70–90 nm) and double-staining with uranyl acetate (8–15 min) and lead citrate (5–10 min). Sections were imaged at 80 kV following standard protocols.

Colony pigmentation was documented after 7-day incubation at 37°C on MGM agar. Growth parameters were determined as follows: (i) Na^+^, Mg²^+^, and pH optima (adapted from Wang et al. (30)); (ii) temperature range: 21–50°C; and (iii) growth kinetics: monitored at OD_600_ using a Tecan Infinite 200 microplate reader. Additional phenotypic tests followed the minimal standards for halophilic archaea (31). Catalase and oxidase activities were determined following the method described by Gutiérrez et al. (32). Gelatin hydrolysis was assayed on MGM agar plates supplemented with 5 g/L gelatin, and hydrolysis was confirmed by flooding the plates with Frazier’s reagent (33). Starch hydrolysis was examined using MGM agar containing 2 g/L soluble starch as the substrate, and hydrolysis was visualized by applying Lugol’s iodine solution (34). Hydrolysis of casein and Tween 80 was examined according to the protocols established by González et al. (35). Production of hydrogen sulfide was tested in MGM broth supplemented with 0.5% (wt/vol) sodium thiosulfate using lead acetate-impregnated filter strips for detection (36).

L-histidine supplementation tests employed final concentrations of 0.05–0.5% in growth media, with experimental design following Li et al. (37).

### DSL9 genome sequencing and assembly

Genomic DNA was extracted from pure cultures of strain DSL9 using the FlaPure Bacterial Genomic DNA Extraction Kit (Genesand, China). The nearly full-length 16S rRNA gene was amplified using the universal primers 21F (5′-TTCCGGTTGATCCYGCCGGA-3′) and 1492R (5′-TACGGYTACCTTGTTACGACTT-3′). The 25 μL PCR reaction consisted of 2× Rapid Taq Master Mix (Vazyme, China) and 10 μM of each primer, using extracted genomic DNA as the template. Amplification was performed under the following conditions: initial denaturation at 95°C for 5 min, 35 cycles of denaturation at 95°C for 15 s, annealing at 55°C for 15 s, and extension at 72°C for 30 s. The resulting amplicons were purified and subjected to Sanger sequencing on an Applied Biosystems 3730XL instrument (Thermo Fisher, USA) by Sangon Biotech (Shanghai).

Whole-genome sequencing was performed using both short-read (Illumina NovaSeq 6000 PE150 platform) and long-read (Oxford Nanopore Technologies) approaches to ensure comprehensive genomic coverage and closure. Reads were quality-controlled with Sickle, version 1.33, available at https://github.com/najoshi/sickle. For genome assembly, short and long reads were co-assembled using Unicycler (38).

### Genome annotation and comparative analysis

Protein-coding genes were predicted using Prodigal (39). Initial functional annotation was performed using DIAMOND (40) against the NCBI non-redundant database, retaining the top 10 hits for each gene. Additional functional characterization was achieved through (i) structural motif identification using MOTIF Search; (ii) protein structure prediction with Foldseek (41); and (iii) remote homology detection via HHpred (42). tRNA genes were identified using tRNAscan-SE (v2.0) with default parameters (43).

Defense systems were characterized using both DefenseFinder (44) and PADLOC (45). Insertion sequences were identified through ISEScan (46) with default parameters. Single XerA recombinases were classified using COGclassifier, with stringent criteria requiring (i) XerD-domain containing proteins and (2) absence of adjacent *xerC* gene within a 20-gene radius.

The horizontal gene transfer network was constructed by identifying shared genes between plasmids using BLASTp (e-value <1e-5), thresholds: ≥98% coverage and identity) and then visualized with Gephi using force-directed algorithms.

The copy number of pHdsl9-1 and pHdsl9-2 plasmid genes in the genome was calculated using MMseqs2 (47) with 50% coverage and 30% identity thresholds for clustering. Singleton clusters in the genome are considered single-copy genes.

Sequence collinearity was calculated and visualized using clinker (48), employing default alignment parameters for comprehensive genome comparison.

### Phylogenetic analysis

The 16S rRNA gene of strain DSL9 was identified using barrnap with default parameters. Related sequences were retrieved from the NCBI database using BLASTn (49), followed by deduplication at 94.5% identity threshold using CD-HIT (50) to obtain representative sequences from each related genus.

The average nucleotide identity of strain DSL9 and other strains of halophilic archaeal strains is performed using fastANI with default parameters (51).

The archaeal phylogeny was reconstructed using a concatenated alignment of 53 marker genes generated by GTDB-Tk (5). The maximum-likelihood analysis was performed with FastTree (52) under default parameters. The tree was visualized using iTOL (53).

XerA recombinase proteins were recruited through homology searches using PSI-BLAST (two iterations, 100 sequences per round) against the nr database. Phylogenetic reconstruction of a 12-conserved-gene concatenated tree, single-protein XerA trees, and concatenated VirB4 and VirD4 protein trees was constructed using FastTree with default parameters.

### Identification and characterization of pHdsl9-3-related plasmids

To identify pHdsl9-3-related plasmid elements, we performed a homology search using DIAMOND to align all pHdsl9-3-encoded proteins against the NCBI nr database. Contigs encoding proteins with significant hits to pHdsl9-3 proteins were retrieved for further analysis. To minimize inclusion of chromosomal sequences in the initial screening, we excluded contigs exceeding 500 kbp in length.

The retrieved contigs were subsequently analyzed using vConTACT2 to identify pHdsl9-3-related plasmid clusters. Based on the functional importance of the T4SS gene cluster in pHdsl9-3 transmission, we established a 30% orthologous fraction (OF) threshold for plasmid inclusion. This threshold was empirically determined to ensure that >90% of recruited plasmids contained the characteristic T4SS cluster.

For identification of conserved genetic elements, protein-coding genes were predicted from all recruited plasmids using Prodigal (39). Genes encoded by pHdsl9-3 were compared against the recruited plasmid gene repertoire using BLASTp. Genes sharing ≥50% coverage and ≥30% identity with pHdsl9-3 homologs were considered commonly encoded, and single-copy genes present in >90% of recruited plasmids were classified as conserved elements.

## RESULTS AND DISCUSSION

### Isolation and phenotypic characteristics of DSL9

Strain DSL9 was isolated from a water sample collected from Dishui Lake with a salinity of 0.04% and exhibited less than 92% of 16S rRNA gene sequence similarity with all known genera of halophilic archaea. This finding strongly indicates that DSL9 represents a novel genus within the class *Halobacteria*.

**Fig 1:**
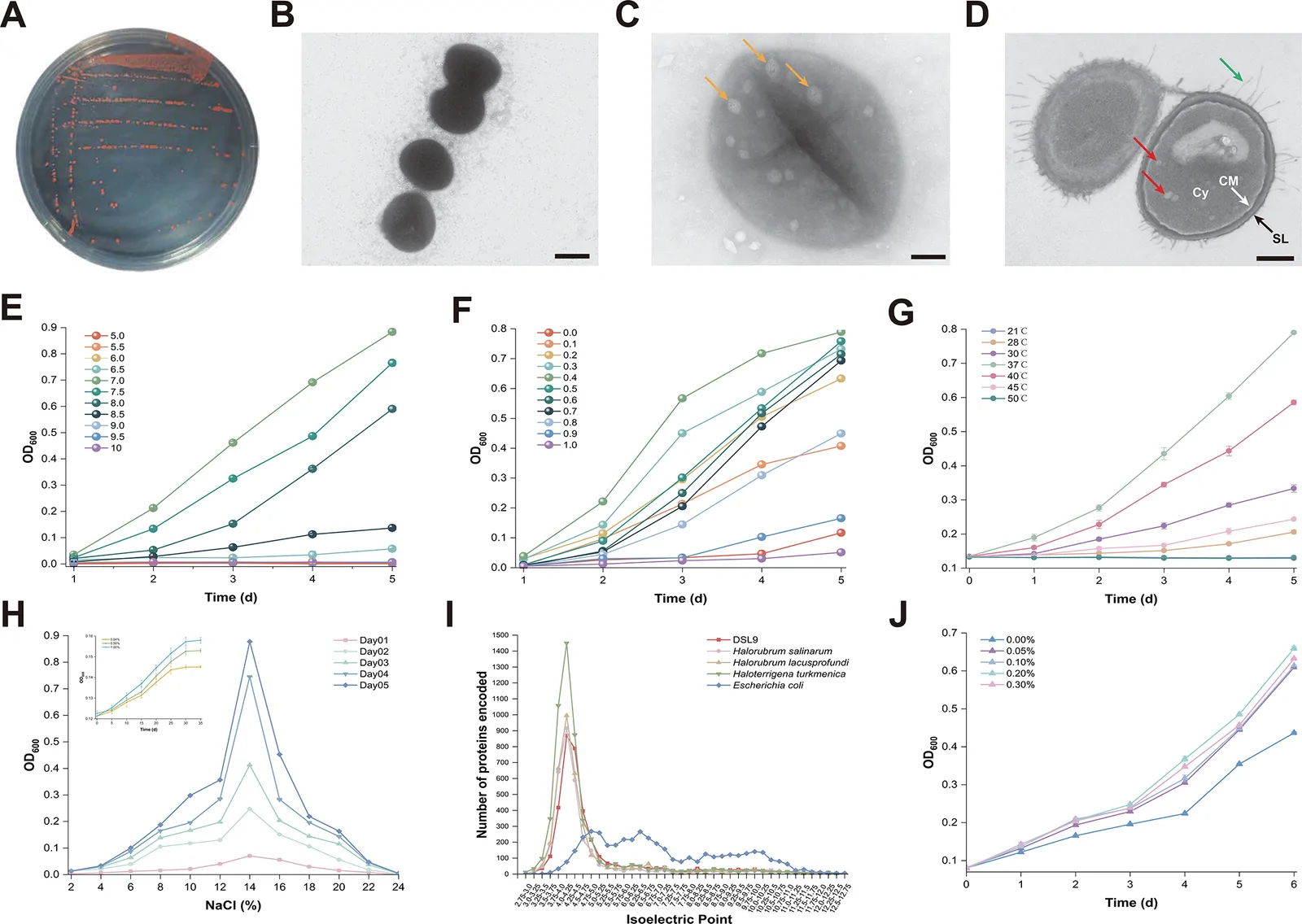
Phenotypic characterization of *Haloliberatus hailidukes* DSL9. (**A**) Colonies of strain DSL9 grown on MGM agar medium at 37°C for 14 days. (**B**) Whole-cell morphology (scale bar: 1 μm). (**C**) High-magnification imaging revealed unique and unknown cell apparatus (yellow arrows) (scale bar: 200 nm). (**D**) Ultrastructural features highlighting putative polyhydroxyalkanoate (PHA) granules (red arrows) and pili structures (green arrow). The cell reveals the typical S-layer, which is superimposing the cytoplasmic membrane and thereby forming a pseudoperiplasm. Cy: cytoplasm; CM: cell membrane (white arrow); SL: S-layer (black arrow) (scale bar: 200 nm). Growth response curves demonstrating the strain’s pH optimum (**E**), magnesium ion requirement (**F**), and temperature optimum (**G**). (**H**) Growth characteristics of isolate strain at various salinities. (**I**) Comparative analysis of protein isoelectric point (pI) distributions. (**J**) Histidine-dependent growth modulation.

Strain DSL9 formed round, moist colonies with vibrant orange-red pigmentation on MGM agar plates (Fig. 1A). The cells were coccoid, measuring 1.0–1.3 µm in diameter, and exhibited consistent morphology in both solid and liquid cultures (Fig. 1B). Cellular shape remained stable across different salinity conditions. Notably, TEM revealed numerous spherical to ovoid net-like structures on the cell surface (Fig. 1C), a feature not previously documented in archaea, which might be linked to freshwater adaptation. Ultrastructural analysis via thin-section TEM identified several distinctive features: (i) dense pili-like appendages surrounding the cells, (ii) inclusion bodies resembling the polyhydroxyalkanoate (PHA) granules of *Haloquadratum walsbyi* (34), and (iii) a typical S-layer overlaying the cytoplasmic membrane, forming a pseudo-periplasmic space (54) (Fig. 1D). Additionally, apparent cell-cell connections were observed, likely mediated by dense pili bundles (55) (Fig. 1D).

Comparative phenotypic analysis distinguished strain DSL9 from related genera in the *Haloferacaceae* family through several key physiological traits (Table 2). The isolate displayed optimal growth under neutral pH conditions (pH 7.0–7.5; range: 6.5–8.5) (Fig. 1E), with a magnesium chloride requirement of 0.4 M (tolerance range: 0–1.0 M) (Fig. 1F) and a temperature optimum of 37°C (growth range: 28–45°C) (Fig. 1G). Biochemical characterization revealed catalase activity but no detectable oxidase production. The strain showed no hydrolytic activity against Tween 80, gelatin, casein, or starch. Notably, DSL9 did not generate H₂S via thiosulfate reduction (Table 2), further differentiating it from related halophilic archaea.

**TABLE 2:** Comparative phenotypic characteristics of strain DSL9 and related cultured *Halobacteriales* genera^*a*^

Characteristic	DSL9	*Halohasta* (6)	*Halonotius* (3, 56, 57)	*Natronocalculus* (58)
Colony pigmentation	Orange-red	Red	Red	Red
Morphology	Coccus-shaped	Rod-shaped	Pleomorphic	Pleomorphic
Motility	–	+	±	–
Cell size (μm)	1–1.3	0.4–0.6 × 1–6	0.7–4.4 × 0.5–6	0.8–2
Lyse in distilled water	–	+	+	NR
Optimum NaCl (%)	14	15–18	18–25	19
Temperature optimum (°C)	37	30–40	37–45	37–40
Optimum pH	7.0–7.5	7.0–7.5	7.0–7.5	8.5–8.8
Optimum Mg^2+^ (M)	0.4	0.5–0.7	NR	NR
H_2_S formation	–	+	–	NR
Starch hydrolysis	–	–	–	–
Casein hydrolysis	–	–	NR	–
Gelatin hydrolysis	–	–	–	–
Tween 80 hydrolysis	–	–	–	–^*b*^
G + C content (mol %)	64.2	62.4–62.9	58–62.7	51.5

^*a*^ NR, not reported. V, variable property in different species of the same genus.

^*b*^ Esterase reported, but further research to confirm Tween 80 hydrolysis was not conducted. Symbols: +, positive; –, negative.

### Low-salinity osmoregulation phenotype of DSL9

Growth analysis across an extreme salinity gradient (0.04–24% NaCl, wt/vol) revealed DSL9’s exceptional osmotic stress response. The strain demonstrated unprecedented hypo-osmotic tolerance for a halophilic archaeon, maintaining cellular integrity even in distilled water (Fig. S1), which is a condition that typically causes immediate lysis in related taxa (Table 2). While growth was largely limited at hypotonic concentrations (0.04–1% NaCl) (Fig. 1H, inset), optimal proliferation occurred at 14% NaCl (Fig. 1H), confirming its halophilic nature.

This remarkable adaptability contrasts sharply with current knowledge of halophilic archaea. Previous studies report that even the most hypo-osmotic-tolerant strains, such as *Haladaptatus* isolates from Japanese salt fields, show only 20–34% survival after 9 days in 0.5% salinity (29). Notably, DSL9 was originally isolated from Dishui Lake (0.04% salinity), representing the first documented case of a halophilic archaeon capable of surviving freshwater conditions. This exceptional phenotype suggests the evolution of novel osmoadaptive strategies, potentially including (i) unique membrane stabilization mechanisms, (ii) specialized osmolyte transport systems, or (iii) previously uncharacterized stress-response proteins.

The strain’s ability to maintain viability across such extreme salinity gradients (0.04–24%) challenges conventional boundaries of archaeal halophily and may represent a new category of extremophilic adaptation.

### Genome features and classification of DSL9

**Fig 2:**
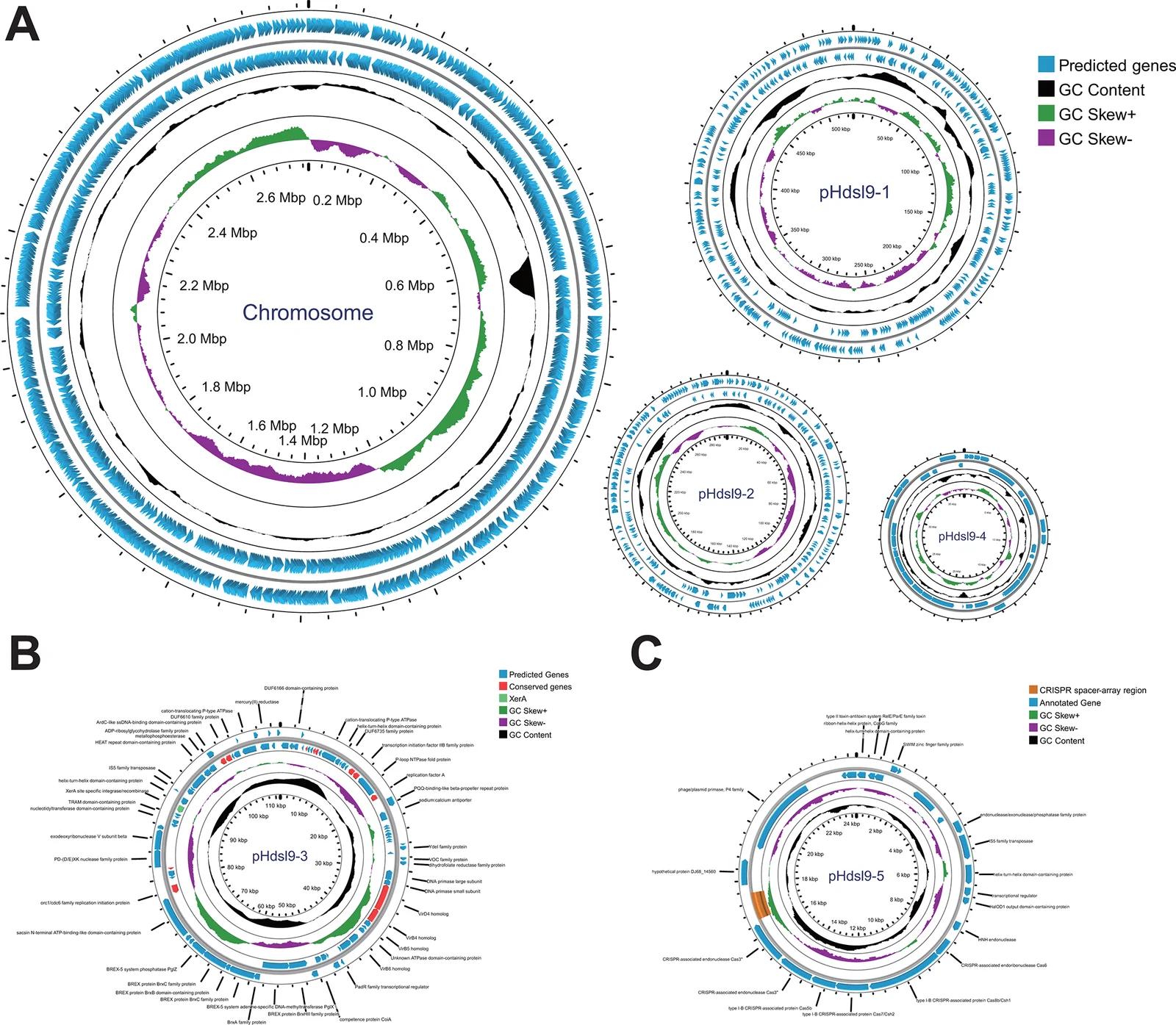
Genomic characterization of *Haloliberatus hailidukes* DSL9. (**A–C**) Circular representation of the complete chromosome and five plasmids of strain DSL9. Outer gray ring represents the genome with predicted ORFs indicated by colored arrows (directionality shown). Inner tracks show the GC content (black), GC skew (purple/green), and scale markers. Plasmid pHdsl9-3 and pHdsl9-5 are annotated with key genomic features in panels (**B**) and (**C**).

The complete genome assembly of DSL9 revealed six circular replicons: one chromosome (2,777,201 bp) and five plasmids (24,278–525,539 bp), collectively spanning 3,735,769 bp with 64.2% GC content (Fig. 2; Table S1). Annotation identified 3,728 protein-coding genes, with all translational machinery (50 tRNAs, 6 rRNAs) located on the chromosome (Tables S1 and S2). The genome contains two identical copies each of 16S, 5S, and 23S rRNA operons.

Comparative genomics revealed several adaptations to salinity fluctuation. A complete *otsAB* operon (UDP-glucose into trehalose) serves as the exclusive compatible solute system, mirroring strategies of euryhaline archaea (7). The TrkA/TrkH potassium transporters and NhaC-type Na^+^/H^+^ antiporters implement the “salt-in” strategy (7). The genome has an overall proteome isoelectric point of median pI = 4.36 (Fig. 1I), a hallmark of halophilic adaptation (59). This highlights DSL9’s unprecedented ability to survive in freshwater while maintaining halophilic characteristics. Meanwhile, the complete histidine catabolism pathway conferred 62% biomass increase rate with 0.2% histidine supplementation (Fig. 1J), suggesting carbon/nitrogen scavenging in nutrient-variable environments (60).

**Fig 3:**
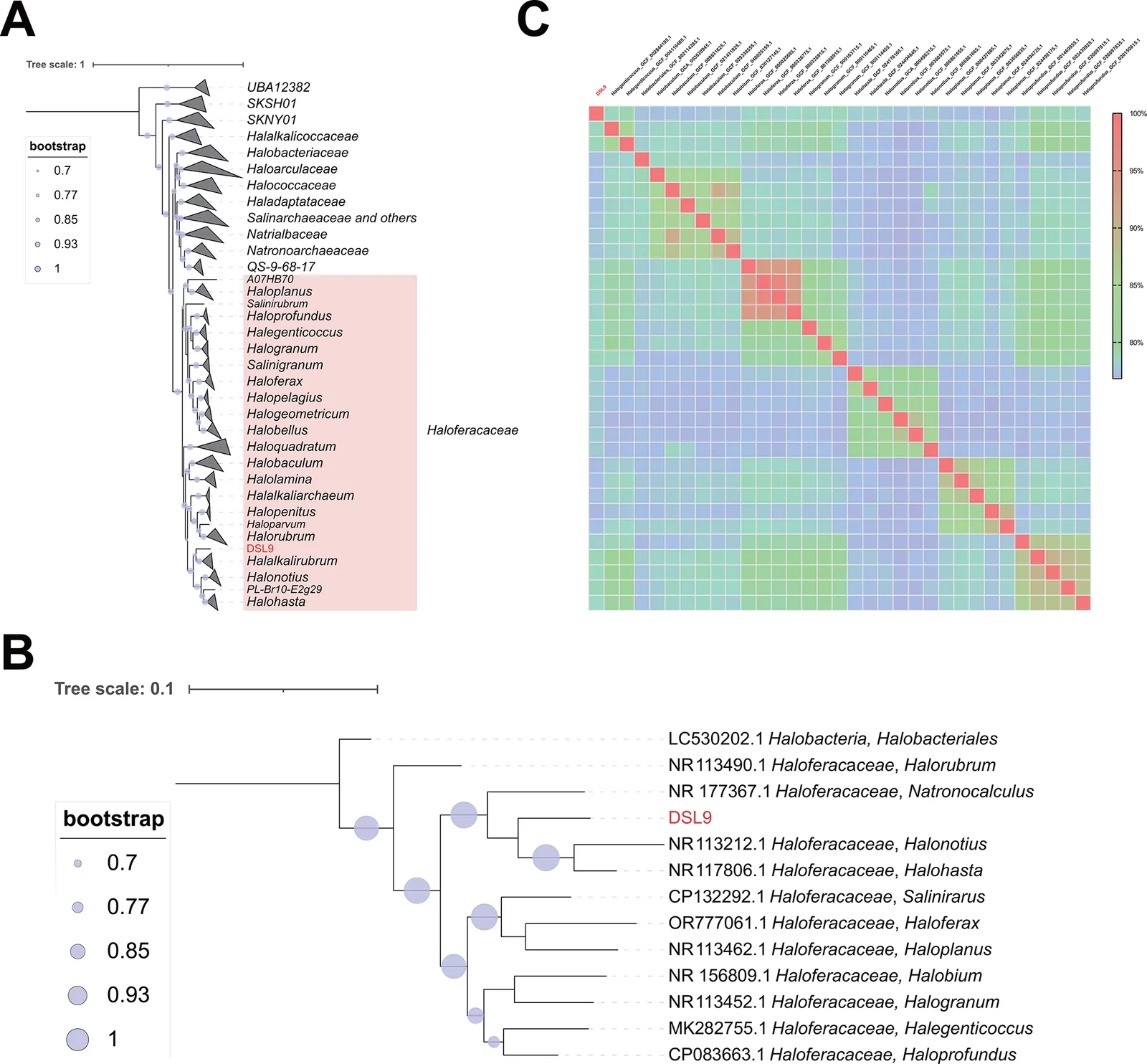
Phylogenetic characterization of *Haloliberatus hailidukes* DSL9. (**A**) Unrooted concatenated 53-marker gene phylogenetic tree of strain DSL9 within the order *Halobacteriales*. (**B**) Maximum-likelihood phylogenetic tree based on 16S rRNA gene sequences, showing the taxonomic position of DSL9 (highlighted in red) among related halophilic archaea. Bootstrap values (>70%) are shown at key nodes. (**C**) Average nucleotide identity between strain DSL9 and most related strains. The colors in the heat map indicate the similarity between the two strains.

Phylogenetic analysis based on a concatenated alignment of 53 conserved archaeal markers (Fig. 3A) robustly positions DSL9 within the family *Haloferacaceae*. Its closest relative in public databases is a member of the genus *Halohasta*, with which it shares only 92.0% 16S rRNA gene identity, which is significantly below the standard genus delineation threshold (~94.5%) (50). Furthermore, both 16S rRNA phylogeny (Fig. 3B) and average nucleotide identity (ANI ≤ 80% with all known *Haloferacaceae* strains; Fig. 3C) confirm that DSL9 represents a distinct lineage, warranting its classification as a novel genus of haloarchaea. This pronounced genetic divergence provides compelling evidence for the proposal of DSL9 as a new taxon within *Haloferacaceae*.

Based on these findings, we propose to classify DSL9 as a freshwater-surviving halophilic archaeon, representing a novel physiological adaptation within this phylogenetic group. The genus is named *Haloliberatus* since the strain is halophilic (halo-) but is found in a freshwater environment and free (-liberatus) of osmotic pressure as necessity with 0.04% salinity and is not lysed in distilled water. The species is given the name *hailidukes* (IPA [haɪlɪdjuːks]) as an anagram of Dishui lake, where the strain originated.

### Plasmids of DSL9

The five plasmids of strain DSL9 were systematically designated pHdsl9-1 to pHdsl9-5, using the prefix “pHdsl9-” (plasmid of halophilic strain DSL9) followed by their corresponding replicon numbers. Strikingly, plasmids pHdsl9-1 and pHdsl9-2 contained exceptionally high proportions of genes that are single-copy within this genome (45.9% and 49.3% of their coding sequences, respectively), suggesting possible specialized adaptive roles. The presence of such large plasmids aligns with genomic features commonly observed in halophilic archaea (13, 28, 61).

Differential abundance analysis revealed substantial variation in plasmid relative copy number, with pHdsl9-3 showing 41–45% reduced coverage relative to chromosomal DNA, while pHdsl9-5 demonstrated 2.6- to 4.4-fold elevated coverage (Table S3). Although such differences may be contributed by sequencing and library construction methods, these marked differences likely reflect either distinct replication control mechanisms governing individual plasmids or environmentally driven selective pressures favoring specific plasmid maintenance.

Notably, plasmids pHdsl9-3 and pHdsl9-5 contained multiple genomic regions exhibiting signatures of recent horizontal gene transfer. These putative horizontal transfer events and their potential functional consequences are analyzed in detail in subsequent sections.

#### Plasmid pHdsl9-3

##### Conservation and evolutionary dynamics of pHdsl9-3 and related plasmids in halophilic archaea

**Fig 4:**
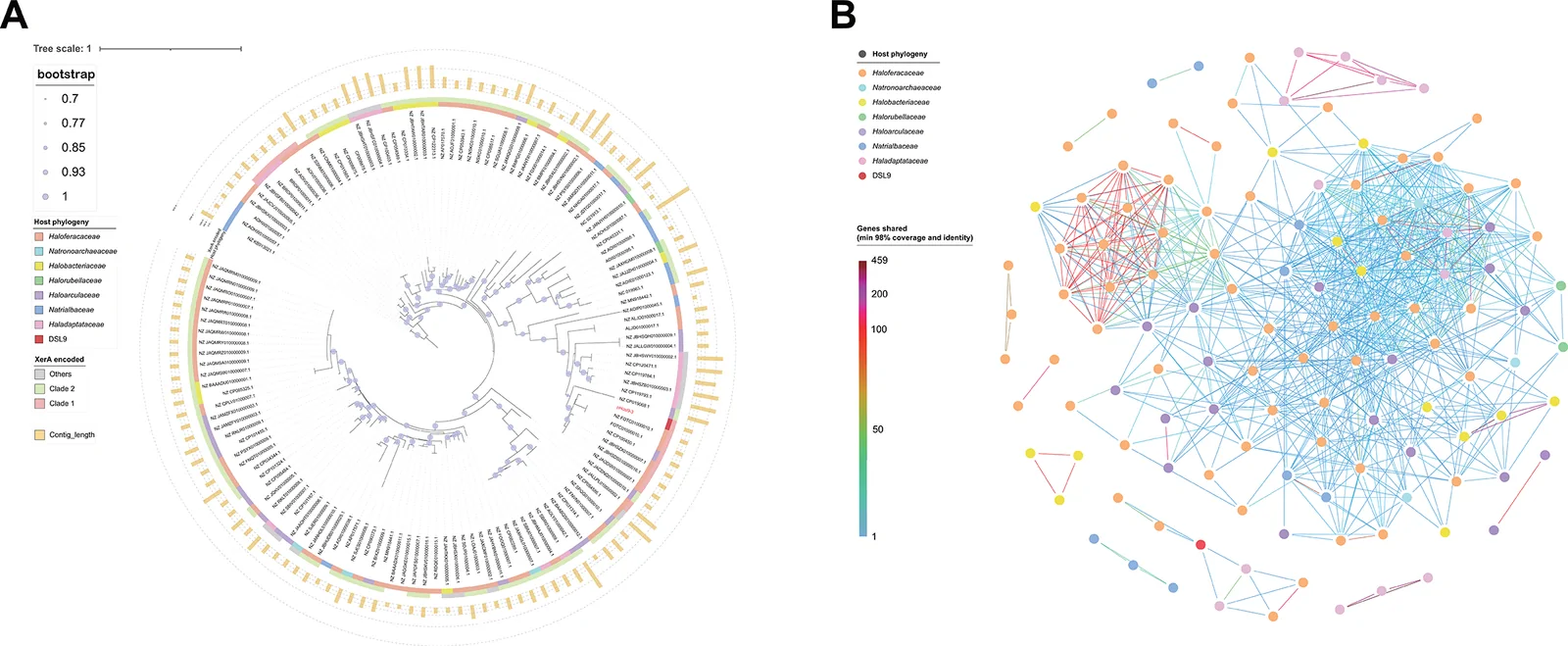
Genomic and evolutionary analysis of pHdsl9-3 and 132 related plasmids. (**A**) Unrooted maximum-likelihood tree (12-conserved-gene concatenated tree) showing evolutionary clustering of pHdsl9-3 and related plasmids. Annotation rings displaying (outer toward inner): (i) plasmid size, (ii) XerA clade affiliation, and (iii) host taxonomic origin. (**B**) Network visualization of genetic exchange among pHdsl9-3 and related plasmids. Nodes represent individual plasmids. Color grading of the edges indicates the number of genes shared, excluding core genes (>98% identity), between the plasmids.

Plasmid pHdsl9-3 (111,311 bp) harbors many genes exhibiting significant sequence similarity (80–97% nucleotide identity) to genes encoded by 132 related plasmids (average length: 160 ± 85 kbp) derived from seven halophilic archaeal families: *Haladaptataceae*, *Haloarculaceae*, *Halobacteriaceae*, *Haloferacaceae*, *Natrialbaceae*, *Natronoarchaeaceae*, and *Halorubellaceae* (Fig. S2). This broad phylogenetic distribution suggests that this plasmid lineage is widespread within the order *Halobacteriales* (Fig. 4A). Notably, the presence of highly similar genes in distantly related hosts, coupled with limited gene sharing among otherwise more divergent plasmids (Fig. 4B), indicates a dynamic evolutionary process involving both whole-plasmid transfers and modular gene exchange. The promiscuous distribution of pHdsl9-3-related plasmids across multiple archaeal families underscores their role as key mobile genetic elements in halophilic communities.

Importantly, these plasmids share a conserved core present in >90% of cases, comprising (i) proteins related to replication (Orc1/Cdc6, single-stranded DNA-binding protein), (ii) transcriptional regulators (TFIIB), (iii) ArdC-like proteins, (iv) a type IV secretion system (T4SS) cluster, and (v) multiple uncharacterized proteins (Table S4). These conserved genomic elements are likely essential for core plasmid functions, including replication, intercellular transmission, and host compatibility (62). The presence of a conserved Orc1/Cdc6 protein and the absence of a canonical Rep protein suggest that plasmid replication likely proceeds via a theta-mode mechanism (61).

Recent research indicates that Orc1/Cdc6 proteins can indirectly inactivate integrase repressors, thereby activating the replicative cycle of pleolipoproviruses (63). Although the specific palindromic motif associated with ORF4 in the proviral activation mechanism was not identified in pHdsl9-3, the potential for its Orc1/Cdc6 homolog to perform a similar or novel regulatory function cannot be excluded, given the abundance of uncharacterized proteins encoded by this plasmid.

##### Adaptive roles of pHdsl9-3 and related plasmids in halophilic archaea

**Fig 5:**
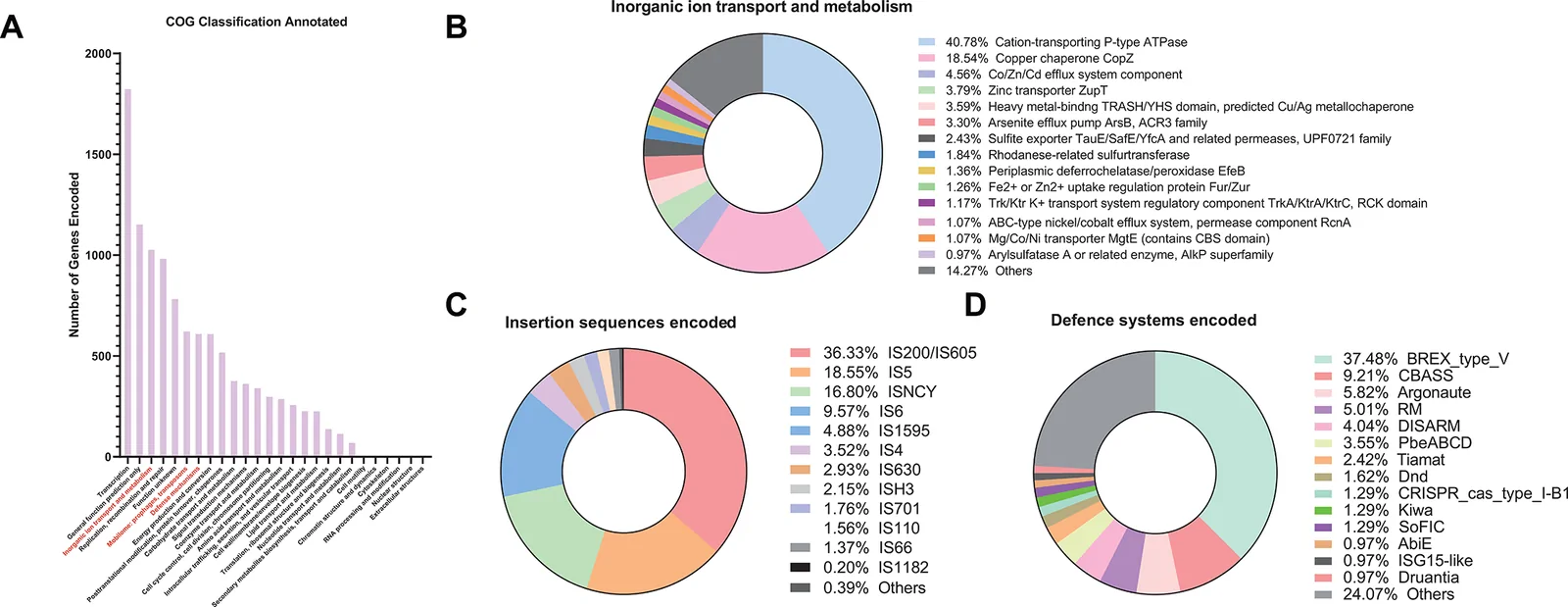
Functional gene categorization of pHdsl9-3-related plasmids. (**A**) Distribution of predicted genes across COG categories. Inorganic ion transport and metabolism, mobilome, and defense mechanisms are highlighted in red. (**B**) Frequency of specific ion transporters. (**C**) Mobile genetic elements. (**D**) Defense system repertoire.

Beyond conserved replication, transcription, and transmission modules, these plasmids predominantly encode genes involved in inorganic ion transport and metabolism, transposable elements, and defense systems (Fig. 5A). Curiously, ZntA—a P-type ATPase responsible for Zn²^+^/Pb²^+^/Cd²^+^ efflux (64)—was the most abundant transporter, detected 420 times across all plasmids (Fig. 5B). A single *Halopenitus salinus* plasmid (NZ_JBHSVN010000002.1) even harbored 14 copies, highlighting its potential importance. This widespread occurrence suggests that these plasmids serve as adaptive reservoirs for heavy-metal detoxification, facilitating rapid genomic plasticity in response to environmental stressors. A similar trend was observed for CszD (Co/Zn/Cd efflux) and ZupT transporters, likely reflecting the elevated metal ion concentrations typical of hypersaline ecosystems.

Mobilome-related elements accounted for 4.0% of plasmid content, with 513 insertion sequences identified among 625 mobilome-associated genes (Fig. 5C). These mobile genetic elements are known to drive genomic rearrangements, potentially accelerating host adaptation (65). Defense systems were dominated by Type V BREX, present in pHdsl9-3 and 40 other plasmids (Fig. 5D; Table S5), underscoring their role in protecting against foreign genetic material.

Notably, the absence of primary metabolic genes suggests that these plasmids function primarily as accessory replicons, conferring niche-specific adaptive advantages rather than essential cellular functions. This interpretation is further supported by the reduced RPKM count of pHdsl9-3 in axenic culture, where diminished selection pressures (e.g., metal stress, foreign DNA invasion) may allow plasmid loss during host proliferation.

##### XerA recombinases in pHdsl9-3 and related plasmids

**Fig 6:**
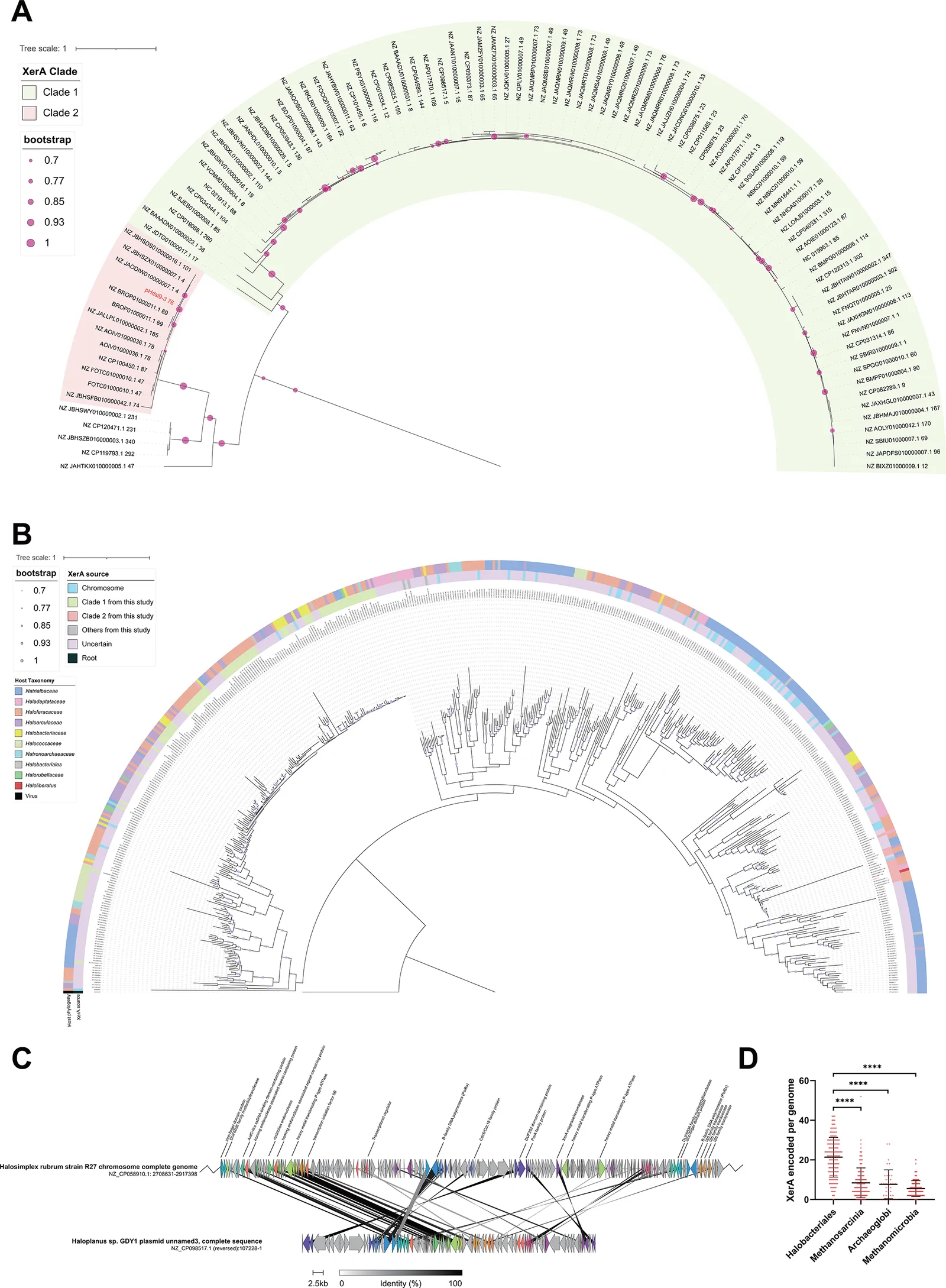
Phylogenetic and genomic context analysis of XerA recombinases in pHdsl9-3 and related plasmids. (**A**) Unrooted maximum-likelihood tree of XerA of pHdsl9-3 and related plasmids. (**B**) Rooted phylogenetic reconstruction of *Halobacteriales* XerA. Host origin was annotated on terminal branches. *Escherichia* phage P1 Cre recombinase is the outgroup. (**C**) Linear genome alignment of the integrated pHdsl9-3-related element found in the genome of *Halosimplex rubrum* strain R27 (top) and the relevant pHdsl9-3-related plasmid from *Haloplanus* sp. GDY1 (bottom). Genes that exhibited more than 80% sequence identity and conserved genes of the pHdsl9-3-related plasmids are designated with labels. (**D**) *xerA* gene distribution across *Halobacteriota*. *P* < 0.0001, denoted by *.

Intriguingly, pHdsl9-3 encodes a single recombinase gene of the Xer family (66), which was present in 75.0% of recruited related plasmids. Phylogenetic analysis shows that these single-domain proteins (containing XerD homology) cluster robustly with the XerA clade (Fig. S3). The absence of a paired gene architecture, combined with this strong phylogenetic placement, leads us to classify them as single-subunit XerA tyrosine recombinases. Phylogenetic analysis of XerA revealed two general clades (Fig. 6A), with other XerA forming scattered lineages, suggesting that those clades may have originated from separate ancestral sources. All recruited plasmids were derived from fully sequenced isolates rather than from metagenomically assembled genomes. Consequently, uncertainties related to sequence provenance are unlikely to confound the findings of this study.

Initial analyses showed that pHdsl9-3-encoded *xerA* shares significant sequence similarity with both plasmid- and chromosome-encoded *xerA*s from various *Halobacteriales* members. This pattern implies that plasmid-encoded *xerA*s may facilitate integration/excision of adjacent genetic material rather than functioning as canonical topoisomerases in genome resolution (22). To investigate this further, we constructed a comprehensive XerA phylogeny incorporating related sequences (Fig. 6B). The tree provides evidence of integration and excision events, although the quality of assembly prevented precise localization of chromosomal or plasmid origin for 71% of *xerA* genes.

Notably, clade 2 *xerA* genes were found in *Haladaptataceae*, *Haloferaceae*, and DSL9, showing a close relationship to chromosomal *xerA* genes in *Haloarculaceae *and *Haladaptataceae*. Clade 1 exhibited greater host diversity, showing a close relationship with chromosomally encoded *xerA* genes from *Haloarculaceae* and *Haloferaceae*. This phylogenetic distribution suggests possible recent plasmid-chromosome exchanges. Supporting this hypothesis, we identified a homologous region between a *Haloplanus* sp. GDY1 plasmid and *Halosimplex rubrum* strain R27 chromosome (Fig. 6C), including the *xerA* gene with 92% identity. The homologous region displays exceptionally high sequence conservation (up to 100% identity) and shares approximately 49% gene content with the *Haloplanus* sp. GDY1 plasmid. This region retained functional elements including helix-turn-helix domains, ZntA ATPase, and various hypothetical proteins, while lacking the T4SS gene cluster. This distinct genomic architecture implies selective evolutionary pressure favoring retention of specific genetic elements following integration events.

The presence of XerA recombinase provides a plausible molecular mechanism for generating the large plasmids typical of this archaeal group. Supporting this hypothesis, comparative genomic analysis reveals that *Halobacteriales* genomes contain significantly more *xerA* homologs than related archaeal lineages, including *Methanosarcina*, *Archaeoglobi*, and *Methanomicrobia* (Fig. 6D).

Despite screening for known Xer recombinase recognition sites (22, 66, 67), none were consistently identified in these plasmids. The absence of a canonical motif raises the possibility that this branch of XerA proteins recognizes distinct, currently uncharacterized sequence motifs.

##### T4SS components in pHdsl9-3 and related plasmids

The core gene cluster contains two major genes encoding proteins with VirB4 and HerA helicase motifs, one of which also carries a VirD4-like motif (Table S6). These conserved components, hypothesized to constitute the core T4SS machinery, are likely responsible for plasmid transmission. The highest nucleotide similarity (91% identity) was found with a chromosomal segment between *Halobacterium salinarum* 91-R6 and pHdsl9-3, with additional matches to various halophilic archaeal plasmids. This pattern suggests both recent transmission events and potential past integration, as discussed below. Notably, 93.2% of pHdsl9-3-related plasmids (sharing >30% orthologous content) encode both VirB4 and VirD4 homologs.

**Fig 7:**
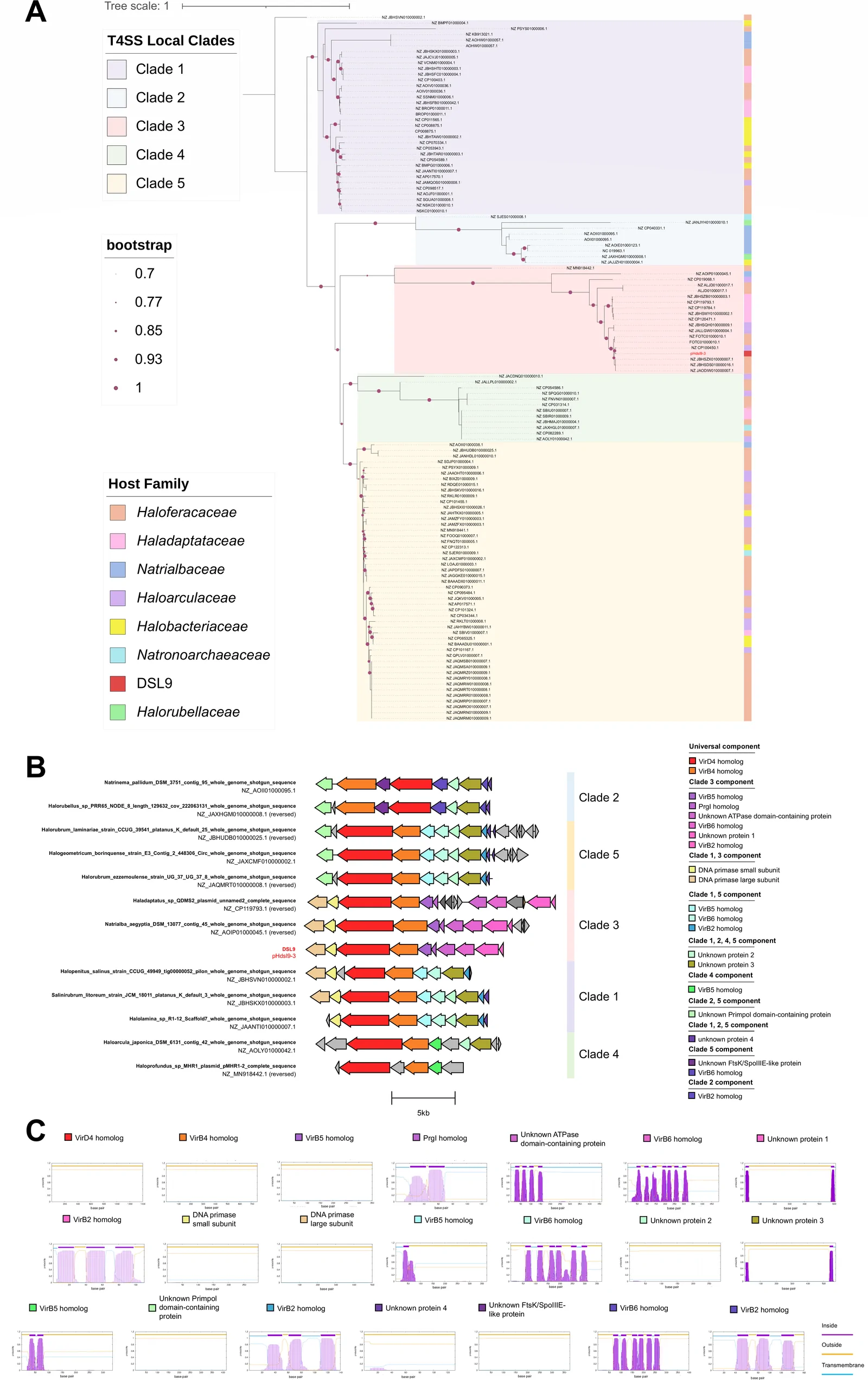
Phylogenetic and structural analysis of T4SS clusters in pHdsl9-3-related plasmids. (**A**) Maximum-likelihood tree based on concatenated VirB4-VirD4 sequences. Five major clades are color-coded. Host taxonomic origin is annotated on terminal branches. (**B**) Linear schematics of T4SS clusters displaying gene inventories for each phylogenetic clade. (**C**) Transmembrane prediction plots for T4SS components. The purple columns are indicative of the predicted transmembrane residues. The blue and orange lines signify the predicted residues located within and outside of the membrane, respectively.

Phylogenetic analysis of concatenated VirB4 and VirD4 sequences revealed five distinct clades (Fig. 7A). While typical T4SSs comprise multiple subunits, only these two showed significant conservation across clades (Fig. 7B). The additional, more variable subunits are shorter transmembrane proteins. Intriguingly, neither VirB4 nor VirD4 possesses predicted transmembrane domains, implying their possible dependence on other membrane-associated proteins for functionality. Upstream of these genes in pHdsl9-3 lie six hypothetical ORFs, five of which contain predicted transmembrane domains (Fig. 7C). Among these, we identified potential VirB5 and VirB6 homologs that may serve as membrane anchors. The pHdsl9-3-specific ATPase domain-containing protein represents the sole transmembrane ATPase in these clades, suggesting a unique functional adaptation.

Structural studies indicate that VirB4 and VirD4 typically form hexameric ATPase complexes that either localize to membranes or associate with membrane proteins to facilitate intercellular transport (68, 69). While some T4SSs employ additional ATPases like VirB11 (70), only clades 3 and 5 in our study encoded extra ATPase-domain proteins beyond VirB4/VirD4. Clade 4 plasmids contained particularly few conserved subunits, raising questions about the minimal functional requirements for these simplified T4SS variants.

Comparative analysis revealed intriguing patterns in subunit composition (Fig. 7B). Despite sharing >30% similarity (as indicated by color-coding), VirB2, VirB5, and VirB6 homologs showed substantial divergence between clades. This pattern suggests either recent homologous recombination events or convergent evolution of functionally similar but phylogenetically distinct components. Such modular variability may reflect adaptive optimization for host range and transmission efficiency, achieved through subunit swapping via homologous recombination or excision-reintegration processes.

### Characterization of pHdsl9-5

The 24,277 bp circular plasmid pHdsl9-5 exhibits two distinctive genetic features: (i) a *primpol* gene encoding a putative primase-polymerase and (ii) an IS5 family transposon (Fig. 2C). The presence of *primpol* suggests an autonomous replication mechanism similar to that observed in pRN1 of *Sulfolobus islandicus* (71), consistent with its stable maintenance as indicated by RPKM values (Table S3). Notably, the replication origin appears distinct from known archetypes, as we identified no characteristic stem-loop structure downstream of *primpol*. The plasmid’s *primpol* gene exhibits significant sequence divergence (<50% identity to database entries), suggesting two possible evolutionary scenarios: (i) long-term plasmid domestication in strain DSL9 as a specialized CRISPR-Cas vector and (ii) representation of an under-sampled plasmid lineage awaiting discovery.

The IS5 transposon displays remarkable sequence conservation, sharing 98% nucleotide identity with both a chromosomal insertion sequence in *Halorussus halophilus* ZS-3 (indicating recent lateral transfer between divergent archaeal families) and a plasmid-borne element in *Natrinema caseinilyticum* ZJ2 (a pHdsl9-3-related plasmid sharing 17.78% orthologous content). This homology supports our hypothesis that pHdsl9-3 may serve as a large mobile genetic element capable of disseminating transposable elements across diverse haloarchaea.

The plasmid pHdsl9-5 contains an incomplete CRISPR-Cas Type I-B system, comprising only the interference module while lacking the canonical adaptation complex (Cas1-Cas2). The 16-spacer CRISPR array shows no significant matches in IMG/VR v4 or nt databases, indicating either targeting of uncharacterized viral or mobile genetic elements, or currently functionally obsolescence due to loss of the adaptation module.

Comparative analysis with known Tn7-like transposon systems (72) revealed no evidence of functional linkage between the IS5 transposon and CRISPR-Cas module: (i) the IS5 element in pHdsl9-5 displays typical inverted terminal repeats (ITRs) exclusively bounding its transposase gene; (ii) no ITR-like sequences flank the CRISPR array or connect both genetic elements. This structural organization differs fundamentally from characterized Tn7-CRISPR associations.

### Conclusion

In this study, we isolated a novel halophilic archaeon, strain DSL9, from Dishui Lake in Shanghai, China. Phenotypic and genomic analyses support its classification as a new species within a new genus, for which we propose the name *Haloliberatus hailidukes*. Genomic sequencing revealed the presence of multiple plasmids. Notably, the large plasmid pHdsl9-3 belongs to a group of plasmids that is widely distributed across diverse families of *Halobacteriales*, highlighting their significant role in driving genetic diversity within haloarchaea. The distinct traits of strain DSL9 provide a valuable model for investigating adaptive strategies to extremely low osmotic pressure. Our findings underscore the importance of further exploring the haloarchaeal mobilome to understand its full ecological and evolutionary significance.

## Data Availability

The complete genome sequence of *Haloliberatus hailidukes* DSL9 has been deposited in the NCBI database under nucleotide accession number CP183369- CP183374.

## SUPPLEMENTAL MATERIAL

Figures S1 to S3 spectrum.03403-25-s0001.pdf Figure S1: DSL9 cell lysis was evaluated in distilled water. Figure S2: Sequence alignment of pHdsl9-3 with other *Halobacterial* members. Figure S3: Phylogenetic analysis of XerA proteins.

Tables S1 to S8 spectrum.03403-25-s0002.xlsx Table S1: General information regarding the genome sequences. Table S2: tRNAs predicted in the chromosomal contig. Table S3: RPKM count for each contig for respective sequencing methods. Table S4: Single-copy conserved genes found in pHfr4. Table S5: All defense system genes predicted from all plasmids included in this study. Table S6: Motifs found in the putative VirB4 and VirD4 homologs found in pHfr4. Table S7: Representative genes used to predict the transmembrane profile. Table S8: COGclassifier result for all genes predicted of the 133 plasmids.

ASM does not own the copyrights to Supplemental Material that may be linked to, or accessed through, an article. The authors have granted ASM a non-exclusive, world-wide license to publish the Supplemental Material files. Please contact the corresponding author directly for reuse.
