# Supplementary material for: Ecological and evolutionary implications of a mobile genetic element-rich haloarchaeon with unique osmotic resilience
Source: Microbiol Spectr. 2026 Jun 30;14(9):e03403-25. doi: 10.1128/spectrum.03403-25 (PMC13532300; doi:10.1128/spectrum.03403-25)
Supplement: Supplementary file 1 [file spectrum.03403-25-s0001.pdf]

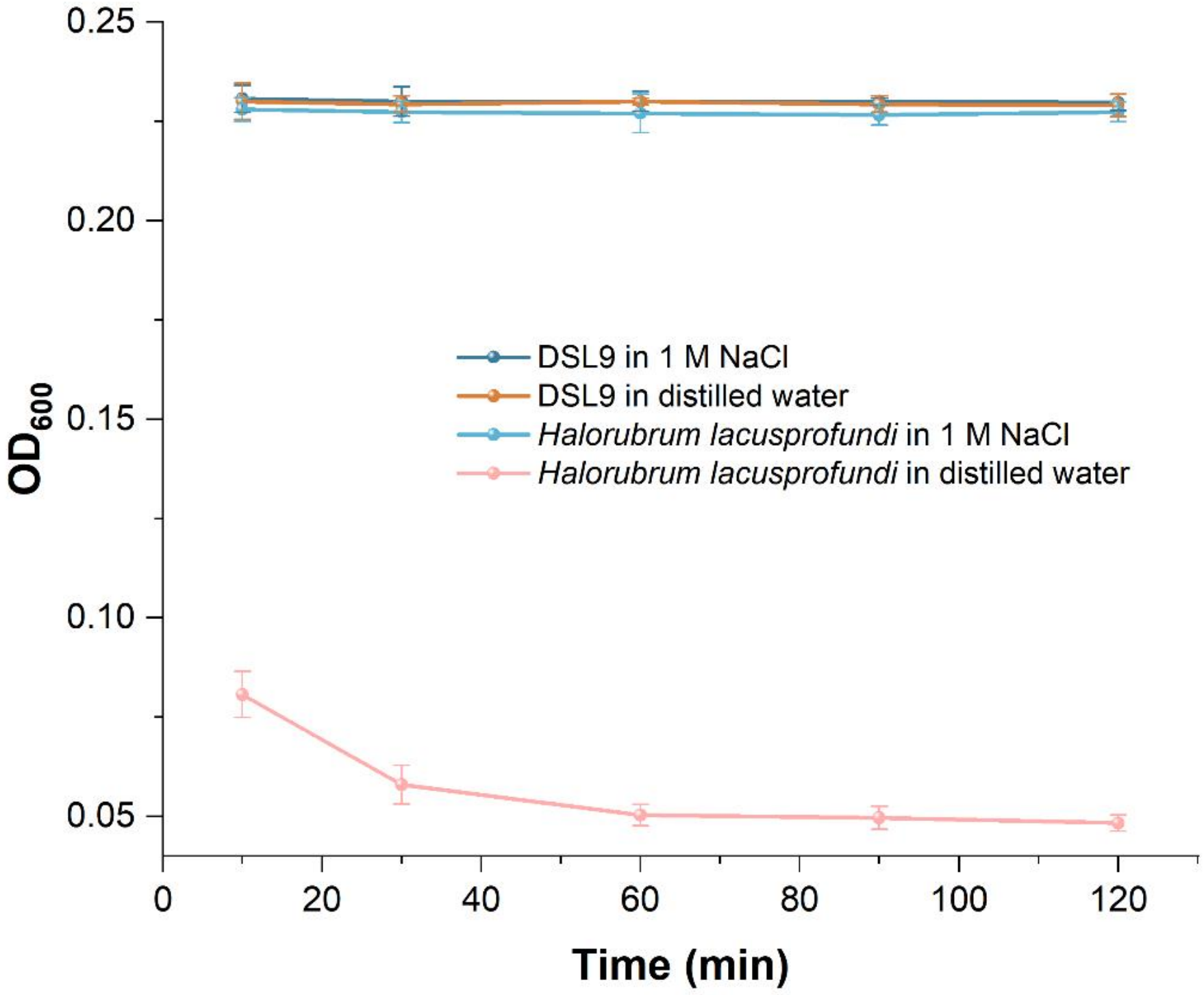

Figure S1. DSL9 cell lysis was evaluated in distilled water, with *Halorubrum lacusprofundi* serving as a halophilic positive control.

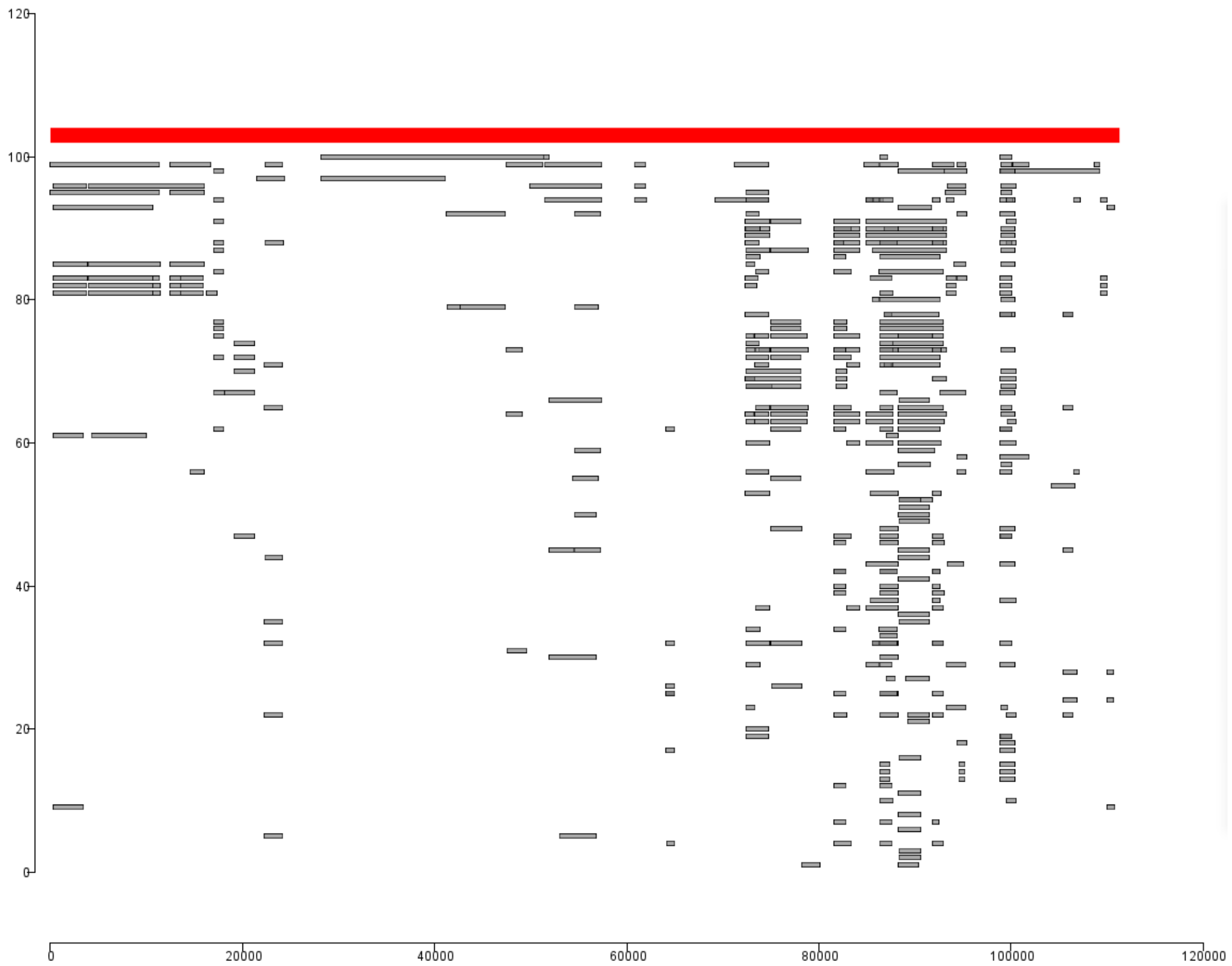

Figure S2. **Sequence alignment of pHdsl9-3 with other halobacterial members**. The red line represents the sequence of pHdsl9-3, with each box beneath indicating a BLASTn hit to other halobacterial sequences. The E-value for these alignments is 0. Width of the boxes reflect the coverage. The X-axis denotes the nucleotide position, while the Y-axis indicates the number of BLASTn hits.

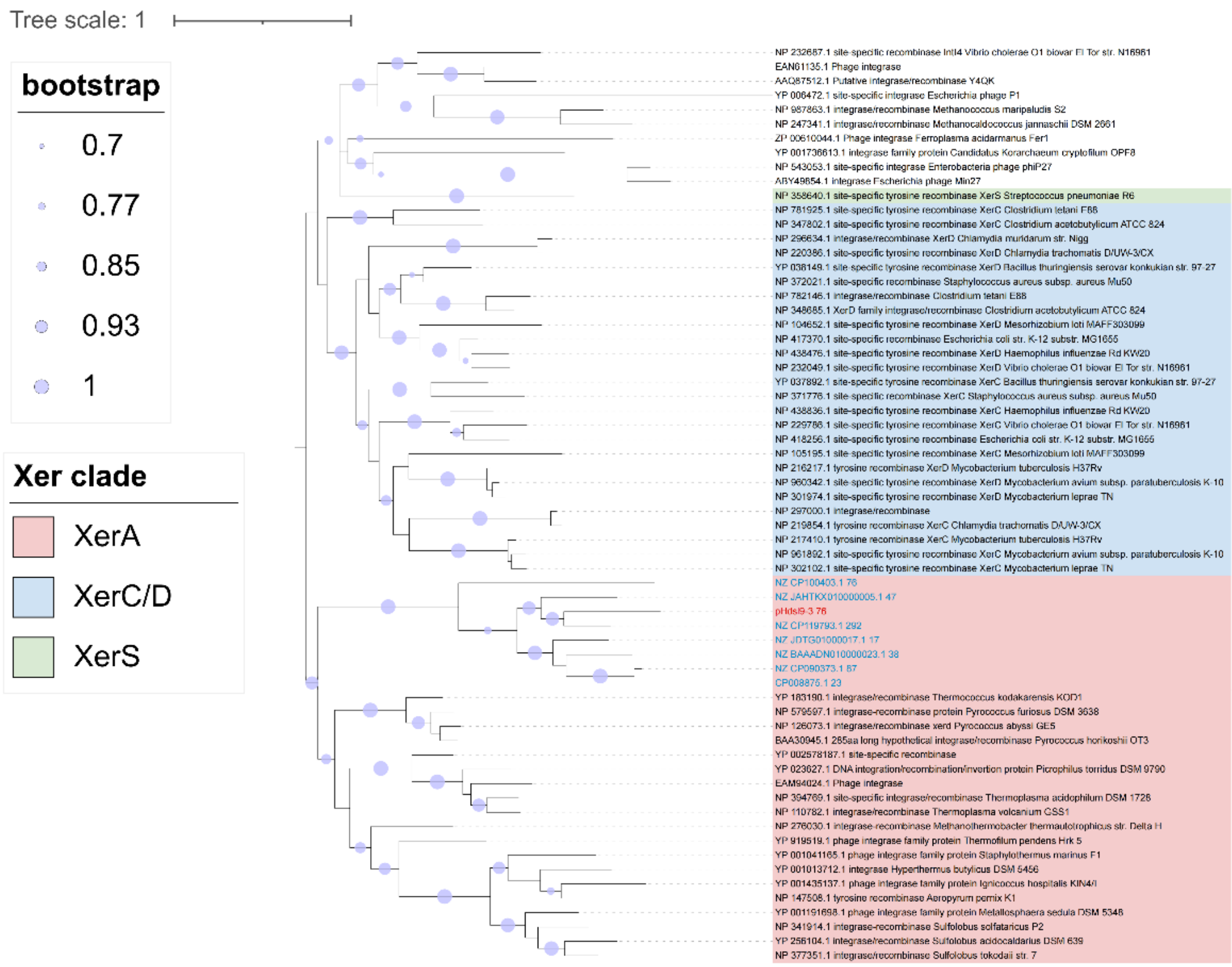

Figure S3. Phylogenetic analysis of XerA proteins. The maximum-likelihood tree shows the relationship between XerA tyrosine recombinases identified in this study (highlighted in light blue and red) and other members of the Xer family. Major Xer clades are indicated, along with bootstrap support values (≥70%) from 1000 replicates.
